# An emerging role of CD9 in stemness and chemoresistance

**DOI:** 10.18632/oncotarget.27021

**Published:** 2019-06-18

**Authors:** Mujib Ullah, Asma Akbar, Avnesh S. Thakor

**Affiliations:** ^1^ Interventional Regenerative Medicine and Imaging Laboratory, Stanford University School of Medicine, Department of Radiology, Palo Alto, California 94304, USA

**Keywords:** CD9, cancer, chemoresistance, stem cells

Cancer drug resistance is a complex phenomenon influenced by drug inactivation, drug target alteration, drug efflux, DNA damage, cell heterogeneity, epigenetic and methylation effects, impairment in cellular communication or any combination of these mechanisms [1]. Development of therapeutic resistance is a persistent problem and is a major cause of cancer deaths worldwide [2]. Cancer cells may acquire resistance to chemotherapy, or may have a high level of resistance through a variety of mechanisms [1]. One example of this is observed in the tumor microenvironment where stem cells can play a crucial role in chemotherapeutic resistance [2]. One method to overcome chemoresistance is to intervene at the level of the interaction between stem cells and tumor cells. Recent studies suggest that CD9 is involved in such cross-talk and and its presence contributes towards chemoresistance [2, 3]. CD9 encodes the transmembrane protein which is involved in tumor cell invasion, apoptosis and resistance to chemotherapy [3]. CD9 has been shown to be involved in a range of cellular activities, including migration, proliferation and angiogenesis, and hence it plays a pivotal role in tumor growth [3, 4]. Previously, it was unclear whether chemoresistance arise from stem cells, tumor cells, or both. Current work has now shown that stem cells can contribute towards tumor cells formation by upregulating CD9, thereby maintaining the tumor cell population [2, 5, 6]. To evaluate molecular changes occurring within tumor cells through CD9 expression, we isolated a hybrid-enriched population of stem and tumor cells using fluorescence-activated cell sorting and performed protein and RNA analysis on these cells [2]. In tumors, CD9 expression is upregulated in hybrid cells that involved in cancer chemoresistance [2]. Although the exact mechanism through which CD9 takes part in chemoresistance is unclear, it is likely that it achieves it through interactions with other proteins. Additionally, CD9 has been implicated in cell fusion, migration, invasion, cancer progression, and chemoresistance [7, 8]. Using the public *Repository for Molecular Brain Neoplasia Data* and the *National Cancer Database* for tumors, we confirmed the prognostic value of CD9 whereby a more than two-fold upregulation was observed with shorter patient survival [6].

CD9 participates in the regulation of the immune system by interacting with B- and T-cell receptors and class I/II MHC antigens [5, 8]. It was found that down-regulation of CD9 by siRNA decreased chemoresistance in different cancers [2, 6]. Moreover, CD9 expression regulated chemoresistance sensitivity by inducing apoptosis and inflammation [5, 7].

Previous studies have shown that CD9 silencing in CD133+ glioblastoma cell lines led to decreased cell proliferation, survival and invasion, which, in turn, can overcome chemoresistance [6, 7]. CD9 is a well-documented marker of stem cells and exosomes whose expression is associated with tumor formation and metastases [3]. However, CD9 expression in various cancer cells is variable and possibly dependent on the biological and genetic background of the cells [3, 7]. Hence, CD9 upregulation may enhance chemoresistance of tumor cells and result in poor survival of cancer patients [6, 7]. Additional *in-vivo* studies are needed to confirm CD9’s involvement in cancer, to determine whether it could be used as a target for potential therapeutic interventions.

The level of expression of the CD9 in tumor cells has been correlated with cancer stem cells proliferation and metastasis in different types of cancer [3, 8]. CD9 knockdown and antibody blockage in breast cancer inhibited stem cell invasion, thereby suggesting that CD9 is required for this process [7]. Remarkably, CD9-deficient breast cancer cells displayed significant alteration of their plasma membrane which might explain the role of stem cells in chemoresistance [3, 8]. As expected, CD9-knockdown suppressed the metastatic capacity of breast cancer cells in mouse xenografts [7–9]. Additionally, it is known that monoclonal antibodies target human CD9 which inhibit tumor growth in xenograft models of pancreatic, breast and leukemia cancer through immune cell activation [6–8]. Furthermore, orthotopic xenotransplantation of CD9-silenced glioblastoma stem cells into nude rats prolonged survival compared to control animals which did not have CD9 silenced [6, 7].

Additional studies have shown that, deletion of CD9 and CD81 in epithelial cells downregulated the expression of SIRT1, which is a key protein that protects against aging and cancer [10]. However, since CD9 expression is also correlated with tumor invasion, downregulation of CD9, rather than complete knockdown, may have positive patient outcomes. [3, 8]. Taken together, these data suggest that CD9 is not only a novel target in cancers which could be exploited for therapeutic uses but it also appears to be a key cancer stem cell marker.

In conclusion, overexpression of CD9 has been observed in many types of cancer which may be linked with enhanced tumorigenicity through overexpression of cytokines (TNF-α, IL-6 and IL-8) and NF-κB activation [7, 8]. Consequently, anticancer drug therapies targeting CD9, such as siRNA, monoclonal antibodies and therapeutic antibodies, could be useful to inhibit the oncogenesis of solid and liquid tumors by reducing chemoresistance.
